# Adenoid cystic carcinoma of the breast in the United States (1977 to 2006): a population-based cohort study

**DOI:** 10.1186/bcr2613

**Published:** 2010-07-23

**Authors:** Bassam Ghabach, William F Anderson, Rochelle E Curtis, Mark M Huycke, Jackie A Lavigne, Graça M Dores

**Affiliations:** 1 Department of Veterans Affairs Medical Center, Medical Service (111), 921 NE 13th Street, Oklahoma City, OK 73104, USA; 2 University of Oklahoma Health Sciences Center, Oklahoma City, OK 73104, USA; 3 Division of Cancer Epidemiology and Genetics, National Cancer Institute, National Institutes of Health, Department of Health and Human Services, 6120 Executive Blvd, Bethesda, MD 20892, USA

## Abstract

**Introduction:**

Adenoid cystic carcinoma of the breast (breast-ACC) is a rare and special type of basal-like tumor for which scant population-based descriptive data exist. We sought to provide new population-based information on breast-ACC incidence, relative survival, and associated cancer risk in the United States.

**Methods:**

Using data from the Surveillance, Epidemiology and End Results Program, we calculated age-adjusted incidence rates (IRs), IR ratios (IRRs), and relative survival for breast-ACC, and standardized incidence ratios (SIRs) for other cancers.

**Results:**

Overall 338 women (IR = 0.92/1 million person-years) were diagnosed with breast-ACC during 1977 to 2006. Blacks had 39% lower IRs than Whites (IRR = 0.61, 95% confidence interval = 0.37 to 0.96), and IRs remained constant over the 30-year period. Ninety-five percent of cases presented with localized stage (*n *= 320; IR = 0.87), and the highest IRs were observed for estrogen receptor (ER)-negative/progesterone receptor (PR)-negative tumors (IR = 0.56). Like other typically ER-negative tumors, age-specific IRs increased until midlife and then plateaued. Five-year, 10-year, and 15-year relative survival was 98.1%, 94.9%, and 91.4%, respectively. The risk of female breast cancer was not increased following (SIR = 0.89, 95% confidence interval = 0.43 to 1.64) or preceding (SIR = 0.71, 95% confidence interval = 0.28 to 1.46) breast-ACC. Similarly, no association was observed for breast-ACC and risk of all other cancers combined, solid tumors, or lymphohematopoietic malignancies.

**Conclusions:**

Breast-ACC among women is characterized by ER-negative/PR-negative expression, rare regional lymph node involvement, a favorable prognosis with excellent survival, and absence of associated cancers. These findings reinforce the importance of tailored treatments for breast-ACC and lend credence to the apparent heterogeneity of basal-like breast cancers.

## Introduction

Adenoid cystic carcinoma (ACC) of the breast (breast-ACC) is a rare basal-like breast cancer [1-3]. Previously termed cylindroma, ACC was initially described by Billroth in 1856 [4] - with the first description of breast-ACC credited to Geschickter in 1945 [5]. The term cylindroma is now used exclusively to describe benign appendageal tumors of the skin. The morphologic appearance of breast-ACC is similar to ACC occurring at other organ sites, including the salivary gland, lung, and skin [1,6-13]. The diagnostic criteria for breast-ACC includes the presence of a biphasic cellular pattern of myoepithelial and epithelial cells (basaloid and ductal) [1,6,14,15]. True to the molecular signature of basal-like tumors [2,3,16], breast-ACC is often estrogen receptor (ER)-negative and progesterone receptor (PR)-negative [17-20] and does not express HER-2-neu [18,20,21]. Clinical studies suggest that breast-ACC is associated with a favorable survival [22-25], thus belying the expected poor prognosis suggested by the absence of hormone receptor expression.

While the epidemiology of other special breast cancer types, including medullary and papillary carcinomas, has been described in population-based studies [26], most information on breast-ACC is derived from case reports and clinical series - with the largest series reporting fewer than 40 cases [22-24,27] - including one population-based study [28]. Reports of breast-ACC have originated from Europe, North America, Australia, and Asia, with the majority describing a female predominance [14,21,23,24,27-39]. Information on race and ethnicity is sparse, and it is uncertain whether a racial predilection exists for breast-ACC [35,40]. In addition, synchronous and metachronous breast tumors and nonbreast tumors occurring with breast-ACC have been described [14,23-25,27,28,32,41,42], but the risk of associated cancers has not been quantified. To gain insight into the epidemiologic features of breast-ACC, we undertook a population-based study in the Surveillance, Epidemiology and End Results (SEER) Program to provide new information on incidence, relative survival, and associated cancer risk.

## Materials and methods

We utilized data from nine cancer registry areas of the SEER Program (SEER-9) that represent approximately 10% of the population in the United States. SEER-9 includes the states of Connecticut, Hawaii, Iowa, New Mexico, and Utah and the areas of Detroit in Michigan, San Francisco in California, Atlanta in Georgia, and Seattle (Puget Sound) in Washington. These nine areas include a racially diverse population representing approximately 9% Whites, 9% Blacks, 13% American Indians/Alaskan Natives, 19% Asians, 46% Native Hawaiians/Pacific Islanders, and 8% of other races in the United States [43].

The SEER Program classifies histology and topography information according to the third edition of the *International Classification of Diseases for Oncology *[44]. We included all cases of microscopically confirmed, invasive (behavior code of/3) ACC (M8200) of the breast (C500-509) diagnosed during 1977 to 2006.

### Incidence

We calculated incidence rates (IRs), IR ratios (IRRs), and 95% confidence intervals (CIs) using the Rate Session in the SEER*Stat software (version 6.5.2; Surveillance Research Program, National Cancer Institute, Bethesda, Maryland, USA) [3]. Incidence rates were age-adjusted to the 2000 US standard population and are expressed per 1 million person-years.

Among females diagnosed with breast-ACC during 1977 to 2006, we assessed IRs according to race (White, Black, other/unspecified), calendar period (1977 to 1986, 1987 to 1996, 1997 to 2006), age (<50, ≥50 years), laterality (right, left, bilateral, unspecified), quadrant (upper inner quadrant (C50.2), lower inner quadrant (C50.3), lower outer quadrant (C50.5), upper outer quadrant (C50.4, C50.6), central/nipple (C50.0-50.1), overlapping (C50.8), not specified (C50.9)), SEER stage (localized, regional, distant, unstaged), and grade (low grade, including grades I and II; high grade, including grade III and IV; unknown). Localized stage includes tumors confined to breast tissue or fat, including nipple and areola (but not skin); regional stage includes tumors that directly extend to local structures (for example, skin, chest wall, muscle, ribs) and/or local lymph nodes (LNs) (for example, axillary, internal mammary); and distant stage includes tumors that have spread to distant organs (for example, bone, lung, adrenal) or distant LNs (for example, cervical, supraclavicular, contralateral axillary/internal mammary). Excluding inflammatory carcinoma, the major change in SEER staging over the study period was that beginning in 2000 infraclavicular LN involvement was considered regional stage whereas it had previously been classified as distant stage.

Information on tumor size and regional LN involvement was not collected in the SEER Program until 1988, and data on ER and PR status did not become available until 1990. We therefore limited analyses of tumor size (≤2 cm, >2 cm, unspecified) and LN status (negative, positive, unspecified) to cases diagnosed during 1988 to 2006, and limited analyses of ER (positive, negative, other/unspecified) and PR (positive, negative, other/unspecified) to breast-ACC diagnosed during 1990 to 2006. Treatment information in the SEER Program is limited to that received with the initial cancer diagnosis (surgery, radiation therapy), and data on subsequent therapy are not collected. Information on chemotherapy and hormonal therapy is not available in the SEER Public Use Database.

Age-specific IRs were calculated according to eight age groups (<15 years, 15 to 24 years, 25 to 34 years, 35 to 44 years, 45 to 54 years, 55 to 64 years, 65 to 74 years, ≥75 years) and depicted on a log-linear scale as previously described [45]. For the purpose of comparison, we calculated age-specific IRs for other microscopically confirmed special types of female breast cancer, including lobular (M8520), mucinous (M8480), tubular (M8211), medullary (M8510), and papillary (M8050, 8260, 8503) carcinomas. According to the SEER Program convention, incidence rates were not calculated for fewer than 16 cases [46].

### Multiple primary cancers

We assessed the risk of subsequent cancer among 2-month survivors of breast-ACC and the risk of subsequent breast-ACC among more than 1.2 million 2-month survivors of all cancers diagnosed between 1 January 1977 and 31 December 2006 using the standardized incidence ratio (SIR) session in the SEER*Stat software. Analyses of multiple primary cancers were limited to females because of the rare occurrence of breast-ACC among males. SIRs or observed-to-expected number of subsequent cancers and exact 95% CIs were calculated by compiling person-years of observation according to age, gender, race, and calendar period beginning 2 months after the diagnosis of cancer to the study end date, date of death, or date of last known follow-up, whichever occurred first. The expected number of subsequent cancers was estimated by calculating cancer IRs according to gender, race, 5-year age groups, and 5-year calendar periods and then multiplying by the person-years at risk.

### Survival

Using the SEER*Stat Survival Session, we estimated 5-year, 10-year, and 15-year relative survival of breast-ACC and 95% CIs using the actuarial method. We included all cases of microscopically confirmed, invasive breast-ACC among women with known age who were diagnosed in SEER-9 during 1977 to 2005 and were actively followed for vital status through 2006. We excluded cases diagnosed among individuals with second or later primary cancers (*n *= 34). There were no cases of breast-ACC diagnosed by death certificate or autopsy, with invalid vital status or dates, or with unknown survival time. Survival rates were not calculated for fewer than 25 cases [46].

## Results

Overall 338 women (IR = 0.92/1 million person-years) (Table 1) and five men were diagnosed with breast-ACC during 1977 to 2006. Owing to the small number of cases among men, further analyses were restricted to women. Mean and median ages at diagnosis were 63 years and 62 years (range 33 to 97 years), respectively, among women. The majority of cases were diagnosed among White women, and Black women had 39% significantly lower IRs than Whites (IRR = 0.61, 95% CI = 0.37 to 0.96). IRs of breast-ACC did not change appreciably over the 30-year study period. Women aged 50 years or older had an 11-fold higher incidence of breast-ACC than younger women (IRR = 11.02, 95% CI = 8.31 to 14.87). Rates did not differ by left-sided or right-sided laterality (IRR = 1.06, 95% CI = 0.85 to 1.32), and there were no cases of bilateral breast-ACC. Most cancers occurred in the upper outer quadrants, with more than 70% lower IRs for breast-ACC occurring in each of the other quadrants. Ninety-five percent of cases presented at localized stage (*n *= 320; IR = 0.87), with regional and distant disease reported rarely. Thirty percent of women (*n *= 102) were treated with radiation as part of initial therapy for breast-ACC, whereas the remaining 70% (*n *= 236) were not.

**Table 1:** Age-adjusted female adenoid cystic carcinoma of the breast incidence rates and incidence rate ratios

	Total			
	
Characteristic	*n*	IR	IRR	95% CI
Total	338	0.92	NA	
Race				
Whites	295	0.97	1.00	Reference
Blacks	20	0.59	0.61	0.37 to 0.96
Other/unspecified	23	~	~	~
Calendar period				
1977 to 1986	79	0.78	1.00	Reference
1987 to 1996	121	1.02	1.30	0.97 to 1.76
1997 to 2006	138	0.96	1.23	0.93 to 1.65
Age				
<50 years	59	0.25	1.00	Reference
≥50 years	279	2.70	11.02	8.31 to 14.87
Laterality				
Right	164	0.45	1.00	Reference
Left	173	0.47	1.06	0.85 to 1.32
Not specified	1	~	~	~
Site				
Upper outer quadrant	105	0.29	1.00	Reference
Upper inner quadrant	29	0.08	0.28	0.18 to 0.43
Lower outer quadrant	23	0.06	0.22	0.13 to 0.35
Lower inner quadrant	14	~	~	~
Nipple/central	28	0.08	0.27	0.17 to 0.41
Overlapping	89	0.24	0.83	0.62 to 1.11
Not specified	50	0.14	0.47	0.33 to 0.67
Stage				
Localized	320	0.87	1.00	Reference
Regional	14	~	~	~
Distant	1	~	~	~
Unstaged	3	~	~	~
LN and size^a^				
LN negative, ≤2 cm	126	0.50	1.00	Reference
LN negative, >2 cm	67	0.26	0.52	0.38 to 0.71
LN positive, ≤2 cm	2	~	~	~
LN positive, >2 cm	3	~	~	~
Other/not specified	46	0.18	0.36	0.25 to 0.51
Hormone receptors^b^				
ER-negative/PR-negative	128	0.56	1.00	Reference
ER-negative/PR-positive	6	~	~	~
ER-positive/PR-positive	21	0.09	0.16	0.10 to 0.25
ER-positive/PR-negative	12	~	~	~
Other/not specified	55	0.24	0.42	0.30 to 0.59

Data from the nine cancer registry areas of the Surveillance, Epidemiology and End Results Program, 1977 to 2006. Incidence rates are age-adjusted to the 2000 US standard population and are expressed per 1 million person-years. Incidence rate ratios are based on unrounded rates. *n*, number of cases; IR, incidence rate; IRR, incidence rate ratio; CI, confidence interval; NA, not applicable; ~, IRs and IRRs not calculated for fewer than 16 cases or other/unspecified race; LN, regional lymph nodes; ER, estrogen receptor; PR, progesterone receptor. ^a^Limited to cases with localized/regional stage diagnosed in 1988 or later. ^b^Limited to cases diagnosed in 1990 or later.

The mean and median tumor size among women diagnosed in 1988 or later was 2.1 cm and 1.8 cm (range 0.1 to 16.0 cm; *n *= 229), respectively. The tumor size was unspecified for 7% of women (*n *= 18). Most women with localized and regional stage breast-ACC had uninvolved LN, with 48% significantly lower incidence of tumors >2 cm compared with smaller tumors (IRR = 0.52, 95% CI = 0.38 to 0.71). Grade was not specified for 61% of cases (*n *= 207), thereby limiting further analysis. The majority of female breast-ACC were ER-negative/PR-negative (IR = 0.56, *n *= 128).

**Figure 1:**
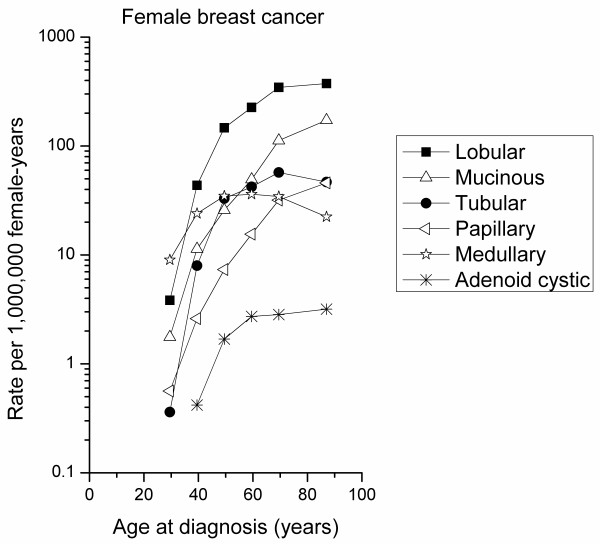
**Age-specific incidence rates of special types of female breast carcinomas**. Data from the nine cancer registry areas of the Surveillance, Epidemiology and End Results Program, 1977 to 2006.

Breast-ACC age-specific IRs increased prominently beginning at ages 35 to 44 years, with a less marked rise in incidence at older ages and an apparent plateau beginning at ages 55 to 64 years (Figure 1). A similar pattern was noted for medullary breast cancer (*n *= 5,973, IR = 16.62), with the exception that IRs decreased more prominently among the oldest age group (≥75 years). In contrast, papillary (*n *= 2,902, IR = 7.72) and mucinous (*n *= 10,404, IR = 27.73) female breast cancer IRs increased exponentially with advancing age. Lobular (*n *= 34,048, IR = 92.52) and tubular (*n *= 5,833, IR = 16.07) carcinomas were characterized by an intermediate age-specific pattern, with IRs increasing rapidly until midlife, followed by a persistent but less steep rise in incidence at older ages.

The overall risk of second-order or higher-order cancers following breast-ACC among females was not increased (SIR = 1.01, 95% CI = 0.72 to 1.38, observed number of subsequent cancers = 40) (Table 2). Subsequent risk of all solid tumors did not differ from that expected in the general population (SIR = 0.94, 95% CI = 0.65 to 1.32, observed number of subsequent cancers = 33), and similarly the risk of any breast cancer was not increased (SIR = 0.89, 95% CI = 0.43 to 1.64, observed number of subsequent cancers = 10). Based on a small number of cases, the risk of lymphohematopoietic malignancies was nonsignificantly increased after breast-ACC (SIR = 1.86, 95% CI = 0.68 to 4.05, observed number of subsequent cancers = 6). No cancers occurred significantly below expectation.

**Table 2:** Standardized incidence ratios of subsequent cancer among 2-month female survivors of first primary breast-ACC

Subsequent cancer^a^	Obs.	SIR	95% CI
All cancers, excluding nonmelanoma skin	40	1.01	0.72 to 1.38
All solid cancers	33	0.94	0.65 to 1.32
Colon and rectum	8	1.43	0.62 to 2.81
Lung and bronchus	7	1.24	0.50 to 2.55
Female breast	10	0.89	0.43 to 1.64
Uterine corpus	3	1.27	0.26 to 3.72
All lymphohematopoietic cancers	6	1.86	0.68 to 4.05
Non-Hodgkin lymphoma	4	2.44	0.66 to 6.24
Acute myeloid leukemia	2	7.24	0.88 to 26.16

Data from the nine cancer registry areas of the Surveillance, Epidemiology and End Results Program, 1977 to 2006. Breast-ACC, adenoid cystic carcinoma of the breast; Obs., observed number of subsequent cancers; SIR, standardized incidence ratio; CI, confidence interval. ^**a**^Limited to cancer sites with >1 case diagnosed among 300 2-month survivors (2,986 female-years; mean follow-up 9.95 years) of first primary breast-ACC. There was one individual with miscellaneous cancer that is not included in the all solid cancers or all lymphohematopoietic cancers categories.

The risk of developing a subsequent breast-ACC following all first cancers and all solid tumors did not differ from that expected in the general population (SIR = 0.86 and 0.91, respectively), and no cases of breast-ACC were reported following lymphohematopoietic malignancies (Table 3). Following a diagnosis of any first primary breast cancer, risk of breast-ACC was not elevated beyond expectation (SIR = 0.71, 95% CI = 0.28 to 1.46). Colorectal and urinary bladder cancers as well as melanoma were associated with nonsignificantly elevated risks of subsequent breast-ACC.

**Table 3:** Standardized incidence ratios of subsequent breast-ACC among two-month female survivors of other first primary cancers

	First primary cancer		Subsequent breast-ACC		
		
Site	*n*	Mean person-years	Obs.	SIR	95% CI
All, excluding nonmelanoma skin	1,239,815	7.04	20	0.86	0.52 to 1.33
All solid	1,122,676	7.24	20	0.91	0.56 to 1.41
Colon and rectum	151,082	6.42	5	1.70	0.55 to 3.96
Melanoma, skin	43,155	9.93	2	2.19	0.27 to 7.91
Female breast	410,448	8.58	7	0.71	0.28 to 1.46
Uterine corpus	90,916	10.48	2	0.69	0.08 to 2.48
Urinary bladder	29,309	7.41	2	2.99	0.36 to 10.81
All lymphohematopoietic cancers	96,222	5.80	0	[*E *= 1.24]	

Data from the nine cancer registry areas of the Surveillance, Epidemiology and End Results Program, 1977 to 2006. Except for the general category of all lymphohematopoietic cancers, the table is limited to cancers with >1 case of subsequent breast-ACC. Breast-ACC, adenoid cystic carcinoma of the breast; *n*, number of first primary cancers; Obs., observed number of subsequent breast-ACC; SIR, standardized incidence ratio; CI, confidence interval; *E*, expected number of subsequent breast-ACC.

The overall 5-year relative survival of breast-ACC was 98.1% with 96 to 97% relative survival observed across all calendar periods studied (Table 4). Five-year relative survival was excellent among women aged <50 years (94.4%) and ≥50 years (99.0%). Among women diagnosed in 1988 or later without LN involvement, 5-year relative survival was 99.1% (*n *= 100) and 89.7% (*n *= 56) among those with tumors ≤2 cm and >2 cm tumors, respectively (data not shown). Overall 10-year and 15-year relative survival exceeded 90%. Women aged <50 and ≥50 years had similar 10-year relative survival (94.4%), but slightly lower relative survival was noted at 15 years for older women (88.9%) than for younger women (94.3%).

**Table 4:** Relative survival among females diagnosed with adenoid cystic carcinoma of the breast

		5-year RS		10-year RS		15-year RS	
				
Characteristic	*n*	%	95% CI	%	95% CI	%	95% CI
Total	285	98.1	79.9 to 99.8	94.9	78.9 to 98.8	91.4	75.0 to 97.2
Calendar period							
1977 to 1986	74	96.8	63.3 to 99.8	95.2	67.1 to 99.4	95.2	67.1 to 99.4
1987 to 1996	109	97.2	81.4 to 99.6	91.0	69.7 to 97.6	85.0	60.7 to 94.9
1997 to 2005	102	96.0	68.5 to 99.6	~	~	~	~
Age							
<50 years	52	94.4	80.3 to 98.5	94.4	80.3 to 98.5	94.3	69.4 to 99.1
≥50 years	233	99.0	6.6 to 100	94.4	72.8 to 99.0	88.9	68.9 to 96.3

Data from the nine cancer registry areas of the Surveillance, Epidemiology and End Results Program, 1977 to 2006. Relative survival is based on cases diagnosed during 1977 to 2005 and followed through 2006. *n*, number of cases; RS, relative survival; CI, confidence interval; ~, insufficient follow-up to calculate survival.

## Discussion

To date the largest breast-ACC studies [14,22-25,27,30,32,36,41], including one population-based study [28], have each included fewer than 40 cases, with some studies including cases diagnosed over a ≥40-year time period [23,24,30]. With more than 300 incident cases of breast-ACC occurring over a 30-year period, our study is the largest reported to date and is the first to describe incidence, survival, and associated cancers. New population-based information includes the predominance of breast-ACC among females and Whites, with the majority of tumors characterized by ER-negative/PR-negative hormone receptor status, localized stage, and absence of regional LN involvement. Breast-ACC IRs remained stable over the 30 years of study, and relative survival was excellent. In contrast to the 60% significantly increased risk of contralateral breast cancer following an initial female breast cancer reported in a previous SEER-based study [47], we did not find an increased risk for breast cancer preceding or following breast-ACC. Similarly, no increased risk was observed for all cancers combined or all solid tumors occurring in association with breast-ACC.

Breast-ACC is a rare malignancy, with approximately one case occurring per 1 million female-years. Based on a substantially larger number of cases, we found a similar mean age at diagnosis as previously reported among 14 histologically confirmed breast-ACC cases diagnosed during 1952 to 1982 in the Connecticut Tumor Registry (mean age 64 years) [28]. Some series have similarly reported mean/median ages of 60 to 66 years at diagnosis [12,22,23,36,42], whereas others have described ages that are nearly one decade younger [17,20,21,24,25,32,34,41]. Breast-ACC often presents with a palpable [15,17,23-25,32,41] and tender [14,15,23,24,37,48,49] mass, which is variably detected on mammography and ultrasound [25,35-37,48,50-52]. Similar to what has been suggested in the literature, we found that tumor size varies widely. Mean tumor sizes of 1.8 to 3.7 cm have been described [12,20,21,23,25,32,42], and not uncommonly the breast mass is noted to have been present several years prior to diagnosis [8,25,30,36,41,49,50,53]. Clinical series report a predominance of centrally located tumors [14,15,19,25,32,51,52,54], nearly equal frequencies of central and upper outer quadrant tumors [15,27,42], or - akin to findings in the SEER Program - tumors occurring primarily in the upper outer quadrant [8,28,34].

Supporting findings in previous reports [15,17-21,27,29,31,35-38,48,50,52,54-57], the majority of breast-ACC in the SEER Program was ER-negative/PR-negative; however, hormone receptor-positive tumors have also been described in a minority of cases [22,23,25,32,34,42,58]. Although information on HER-2-neu is not available in the SEER Program, other series have uniformly reported HER-2-neu-negative status in breast-ACC [18,20,21,23,31,34,37,38,42,50,52,55,56]. Like HER-2-neu negativity, c-kit expression is typically a poor prognostic feature that also characterizes breast-ACC [18,21,29,31,34] as well as medullary breast cancer, another basal-like breast cancer [59,60]. In contrast to what might be expected with ER-negative, PR-negative, HER-2-neu-negative (triple-negative) breast cancers, the majority of cases of breast-ACC in the SEER Program rarely involved regional LN and most were associated with excellent survival. These findings support clinical reports that largely [12,20-25,29,31,32,34-38,41,42,48,50-52,55,56], but not exclusively [21-24,34,42,49], describe absence of LN involvement and excellent survival [22,24,25]. Metastatic breast-ACC has been rarely reported at initial diagnosis [22], and only one case was observed in the SEER Program over a 30-year period.

The mainstay of therapy for breast-ACC has been surgical excision with or without local radiotherapy, with infrequent use of chemotherapy and hormonal therapy [21-24,38,41,42,52,55]. Single or multiple recurrences may occur years after initial diagnosis [8,11,13,20,21,23,27,32,33,39,40,49], and a similar pattern of recurrence has been observed for other triple-negative breast cancers [61].

Similar to the IR pattern previously described for medullary breast cancer, a predominantly ER-negative tumor [26], we found an early-onset incidence pattern for breast-ACC - a rapid rise in incidence at young ages with falling or flattening incidence rates after midlife. Early-onset breast cancer incidence patterns suggest a prominent etiologic role for hormonal influences that occur early in reproductive life [26]. This is, at least in part, a plausible explanation for the markedly higher incidence of breast cancer among females (IR = 132.5/100,000 woman-years) than males (IR = 0.2/100,000 man-years) [62]. In contrast, late-onset incidence patterns - as exemplified by papillary and mucinous breast cancers, typically ER-positive cancers of presumed luminal origin - are characterized by a progressive rise in incidence with advancing age, possibly due to lifelong carcinogenic events and/or exposures [16,26]. Interestingly, papillary carcinomas are among the most common special breast cancer types in males, whereas medullary carcinomas are among the least common [62]. Lobular and tubular breast cancers have age-specific curves that are intermediate between early-onset and late-onset patterns and are postulated to represent a bimodal, mixed population of early-onset and late-onset cancers [26].

The incidence of breast-ACC remained stable during the 30-year study period with no notable rise after the widespread use of mammography beginning in the 1980s. Systematic mammographic screening in the United States has largely resulted in an increase in breast cancer IRs among women ≥50 years of age and in a shift to older age at diagnosis [63,64]. Since breast-ACC is characterized by an early-onset age pattern, this may explain, at least in part, why the introduction of mammographic screening has not apparently influenced temporal patterns. In addition, breast-ACC has been reported to be variably detected on mammogram [25,35-37,48,50-52], thereby potentially contributing to the lack of rise in IRs with the introduction of mammographic screening. Temporal patterns should also be interpreted in the context of the rarity of breast-ACC, such that the relatively small numbers of cases may have limited our ability to detect significant changes in IRs.

Breast-ACC has been described worldwide but race is rarely specified among reports in the literature [35,40]. We found a significantly lower incidence of breast-ACC among Blacks than Whites, which contrasts with the twofold higher incidence of medullary breast cancer described among Blacks compared with Whites [3,26]. These findings suggest that racial differences in susceptibility exist within subtypes of basal-like breast cancers.

Synchronous and metachronous *in situ *and invasive carcinomas have been described in the ipsilateral breast as well as in the contralateral breast in women with breast-ACC [12,14,23-25,31,32,36,42]. Notably, when breast-ACC occurs in conjunction with breast cancer of another histologic subtype, the prognosis is that of the other subtype [7,12,41]. Nonbreast cancers - including lymphoma [24], ocular melanoma [25], and cancers of the ovary [28], endometrium [24], supraglottis [24], kidney [24], lung [27], and skin [42] - have been described among patients with breast-ACC. Based on 18 cases of breast-ACC, Millar and colleagues found a subsequent cancer rate of 13%, 19%, and 26% at 5 years, 10 years, and 15 years, respectively [24]. We did not find a significantly increased risk of all cancers combined, or cancers of the breast or other sites prior to or following breast-ACC when rates were compared with the US general population.

The strengths of our study include the relatively large number of breast-ACC in a population-based setting, which avoids biases inherent to clinical series. Despite the size of our study, however, one limitation is that too few men were diagnosed with breast-ACC to allow calculation of IRs or survival rates. While SEER-9 represents approximately 10% of the US population, our findings may not be generalizable to women in the entire country nor to other non-US populations, particularly if the racial composition differs. Although we included only microscopically confirmed cases, we did not undertake a pathology review and we cannot exclude the possibility of misclassified cribriform carcinoma, benign collagenous spherulosis, or other entities included in the differential diagnosis of breast-ACC [7,12,15,20,41,42,65,66]. Reassuring, however, is that a pathology review of breast-ACC undertaken in the Connecticut Tumor Registry during 1952 to 1982 found 79% accuracy for cases diagnosed after 1974 in contrast to 23% accuracy for cases diagnosed prior to 1974 [28].

Another limitation of our study is that information on hormone receptor status was derived from numerous laboratories using different measurement techniques with varying definitions of positive receptor status. Nevertheless, substantial agreement has been reported between centralized laboratory and SEER registry classification for ER-positive/PR-positive and ER-negative/PR-negative breast cancer subtypes [67]. Without a formal pathology review, it is not possible to determine whether the ER-positive and PR-positive breast-ACC cases we observed are misclassified by histology and/or receptor status or represent a true variant of breast-ACC. Notably, hormone receptor-positive breast-ACC has been reported in several clinical series [22,23,25,32,34,42,58]. We were also unable to assess breast-ACC recurrence rates because the SEER Program does not collect information on recurrent disease. Finally, despite the relatively large size of our study, it is possible that significant cancer associations may have gone undetected because of the small absolute number of breast-ACC cases.

## Conclusions

The present study is the largest of breast-ACC reported to date and the first to describe population-based incidence, survival, and associated cancer patterns. Supporting previous descriptions from clinical series, we found a predominance of breast-ACC among women characterized by ER-negative/PR-negative hormone receptor status, rare regional LN involvement, and excellent survival. An increased risk of breast cancer or other cancers overall was not observed in association with breast-ACC; however, larger studies will be needed to confirm our findings. Awareness of the favorable clinical behavior of breast-ACC is important, and these findings emphasize the need for clinicians to balance the risks and benefits of cytotoxic therapy given the excellent long-term survival. Future studies may consider pooling cases of breast-ACC to develop additional insight into the molecular pathogenesis, etiology, and best treatment approaches of this rare basal-like breast cancer.

## Abbreviations

ACC: adenoid cystic carcinoma; breast-ACC: adenoid cystic carcinoma of the breast; CI: confidence interval; ER: estrogen receptor; IR: incidence rate; IRR: incidence rate ratio; LN: lymph node; PR: progesterone receptor; SEER: Surveillance: Epidemiology and End Results; SEER-9: nine cancer registry areas of the Surveillance: Epidemiology and End Results Program; SIR: standardized incidence ratio.

## Competing interests

The authors declare that they have no competing interests.

## Authors' contributions

BG and GMD participated in the conception and design of the study. GMD analyzed the data. All authors participated in interpretation of the data. BG and GMD drafted the manuscript. All authors participated in critical revisions of the manuscript for important intellectual content. All authors approved the final version of the manuscript.
